# Spontaneous Group Synchronization of Movements and Respiratory Rhythms

**DOI:** 10.1371/journal.pone.0107538

**Published:** 2014-09-12

**Authors:** Erwan Codrons, Nicolò F. Bernardi, Matteo Vandoni, Luciano Bernardi

**Affiliations:** 1 Department of Public Health and Neuroscience, University of Pavia, Pavia, Italy; 2 Department of Brain and Behavioural Science, University of Pavia, Pavia, Italy; 3 Department of Psychology, McGill University, Montréal, Québec, Canada; 4 Department of Internal Medicine, University of Pavia, Pavia, Italy; 5 Folkhälsan Research Center, University of Helsinki, Helsinki, Finland; IIT - Italian Institute of Technology, Italy

## Abstract

We tested whether pre-assigned arm movements performed in a group setting spontaneously synchronized and whether synchronization extended to heart and respiratory rhythms. We monitored arm movements, respiration and electrocardiogram at rest and during spontaneous, music and metronome-associated arm-swinging. No directions were given on whether or how the arm swinging were to be synchronized between participants or with the external cues. Synchronization within 3 groups of 10 participants studied collectively was compared with pseudo-synchronization of 3 groups of 10 participants that underwent an identical protocol but in an individual setting. Motor synchronization was found to be higher in the collective groups than in the individuals for the metronome-associated condition. On a repetition of the protocol on the following day, motor synchronization in the collective groups extended to the spontaneous, un-cued condition. Breathing was also more synchronized in the collective groups than in the individuals, particularly at rest and in the music-associated condition. Group synchronization occurs without explicit instructions, and involves both movements and respiratory control rhythms.

## Introduction

Human beings coordinate movements with each other: marching in step, dancing, singing and playing music in unison are synchronous ritualistic activities belonging to different cultures [1]. In some instances, synchronization seems to develop spontaneously [2], to the point that when two people share visual information they immediately tend to coordinate their movements even when they are instructed to try to be intentionally uncoordinated [3]. A tendency to synchronize is found even in monkeys [4], and in humans starts before birth, as evidenced by high cardiac and respiratory synchronization between mother and fetus in humans [5]. The dynamical theory of interpersonal coordination has provided a general framework to understand similar phenomena in terms of self-organizing dynamical systems [6], as a “free interplay of forces and mutual influences among components tending toward equilibrium or steady states” ([7], pp. 6).

Interpersonal synchronization appears as an important phenomenon, as it seems to promote interpersonal cooperation [8]–[9]. For example, previous studies showed that synchronized movement increases relationship quality and prosocial behaviours [9]–[12]. However, the mechanisms that allow such a leap, from motor synchronization to the feeling of interpersonal bonding, are still poorly understood. An interesting candidate to look at is the physiology of the autonomic nervous system. Dyadic and collective activities requiring temporally coordinated actions are likely to be associated with specific cardiac and respiratory patterns that synchronize within and between people. Recently the synchronization of respiration and heart rate variation has been investigated between persons during choir singing [13] and between two individuals within a romantic relationship [14]. Collective rituals have also shown to evoke synchronized cardiac arousal over time between active participants and bystanders [15]. However, no study up to date has investigated the physiological correlates of performing a simple action in a group of individuals, in the absence of synchronization instructions, intimate relationship or shared goals.

The present study investigates the degree of interpersonal synchronicity in autonomic nervous system during spontaneous motor behaviour within a group. A simple arm movement was performed in a group that did not receive any explicit instructions about synchronization. To control for spurious synchronization in the group due to merely experiencing a movement intervention, the protocol was repeated in a separate group of similar participants who were studied individually. Our main goal was to test whether spontaneous movement within a group was capable of creating interpersonal synchronization of motor, cardiac and respiratory dynamics. We expected synchronization between participants to be higher within the group than within individual subjects, and during movement than during rest, for both respiration and heart rate. We also assessed the effect of an external, auditory rhythm on group synchronization, and to this end we contrasted silent execution with metronome-associated and music-associated execution, always in the absence of explicit instruction about synchronization. Finally, we investigated potential learning effects over 2 days of testing in the collective measurement group.

## Methods

### Participants

The study involved 60 young healthy university students (average age 21.6±1.1, mean ± standard deviation). Although the participants belonged to the same first-year motor-science class (among 115 registered students) they neither had close interpersonal connections nor shared common sports or other activities except those required for their graduation program. The anthropometric characteristics of the participants are shown in Table 1. None of the participants were taking any medications.

**Table 1:** Anthropometric data of the study participants.

	Collect 1 (n = 10)	Collect 2 (n = 10)	Collect 3 (n = 10)	Indiv 1 (n = 10)	Indiv 2 (n = 10)	Indiv 3 (n = 10)
**Sex (Male, Female)**	7 M, 3 F	7 M, 3 F	1 M, 9 F	7 M, 3 F	5 M, 5 F	8 M, 2 F
**Age (years)**	20.6±0.8	21.6±1.1	20±3.2	22.2±1.1	22.9±4	22.4±2.7
**Weight (kg)**	67.1±7.1	64.1±11.9	62.1±5.6	64.8±6.5	65±11.1	73.6±9.1
**Height (cm)**	177.4±9.9	170.6±9.4	168.1±4.6	173.8±5.7	174.3±11	177.5±8.5
BMI (kg*m^−2^)	21.3±1.4	21.8±2.4	22.±2.3	21.5±2	21.2±1.7	23.4±2.6
Mets (h*week^−1^)	26.4±9.2	24.3±21.1	13±7.8	29.2±13.8	42.4±34.3	19.7±13.4

Values are mean ±SD. Collect: Collective measurement group. Indiv: Individual measurement group. BMI: body mass index. Mets: energy expenditure per week.

### Ethics Statement

The individuals in this manuscript gave written informed consent, as outlined in the PLOS consent form, to participate in the study and to publish their case details. The Ethics committee of the University of Pavia, Italy approved the study protocol.

### Equipment

**Figure 1:**
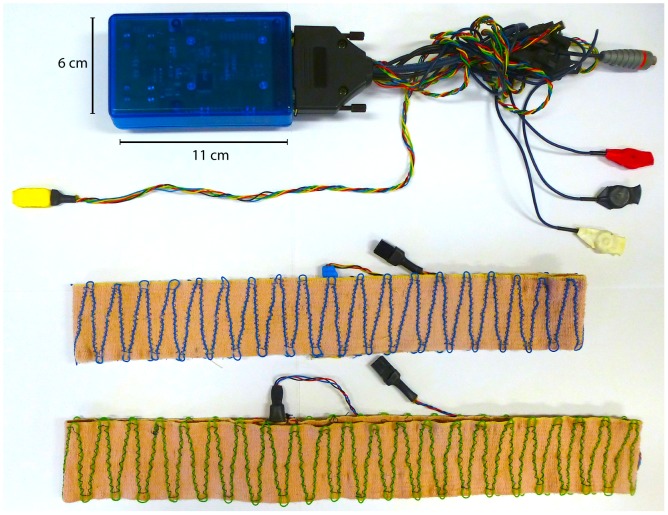
Portable experimental device. The device acquired electrocardiogram, respiratory excursion and arm movement, at the frequency of 400 Hz/channel. The data were acquired simultaneously from the ten participants and synchronized through a built-in XBEE radio module and saved on SD card.

Measurements from each participant were obtained through a special microprocessor-driven portable unit designed and built in our laboratory, shown in Figure 1. Participants wore their unit clipped to their trousers, so that the device did not interfere with their movements. This unit was capable to obtain one electrocardiogram from 3 standard thoracic leads (to obtain a bipolar D2 derivation, in order to record a well defined positive R wave), respiratory excursions from the abdomen and from the chest using the technique of inductive plethysmography (which consisted in positioning 2 highly flexible elastic belts around the upper chest and the abdomen just below the xyfoid), and the movement of one arm by a 3-axis accelerometer. Each unit included a 12-bit data acquisition system which digitized the data at the frequency of 400 Hz/channel on a total of 12 channels. In order to obtain synchronization of all the recordings at the same time, each unit was equipped with an XBEE (Digi International Inc., Minnetonka, MN, USA) radio module (more information about XBEE units and about how to obtain a network of units can be found at: http://www.digi.com/products/wireless-wired-embedded-solutions/zigbee-rf-modules/point-multipoint-rfmodules/xbee-series1-module#overview, and: http://www.digi.com/pdf/ds_xbeemultipointmodules.pdf). One additional radio module was connected to a Macintosh (MacBook Air) portable laptop which served as “coordinator”. This constituted a wireless network that could perform several operations on either individual units (e.g., checking signal quality, adjusting the gain on one signal if needed), by linking the laptop-coordinator to one specific external module or to all units simultaneously. Each unit stored the data on a Secure Digital memory card of 2 GB memory as binary files. A specific code automatically named each file in order to easily identify each participant and each recording. Accordingly, recordings could be started and stopped from the laptop to all units simultaneously, hence providing synchronized files with corresponding file names. Once the acquisition was completed, data were uploaded on the computer and evaluated. Tests on synchronization showed that the actual time difference in a 4 minute recording was below 20 ms across all units.

### Experimental procedure

The subjects were randomly assigned to 6 groups of 10 participants each: 3 “collective measurement groups” and 3 “individual measurement groups”. The two types of group underwent the identical sequence of recordings, but while in the collective measurement groups the measurements were done simultaneously in all participants (see description below), in the individual measurement groups each participant was studied alone, with no other participant present in the room. The participants in the individual measurement groups were studied only once, whereas in the collective measurement groups the protocol was repeated twice to assess whether the group induced a learning effect in synchronizing the individual behaviour.

In the collective measurement groups the participants seated in circle, at the same distance one from each other. The experiment was conducted in a quiet room.

**Figure 2:**
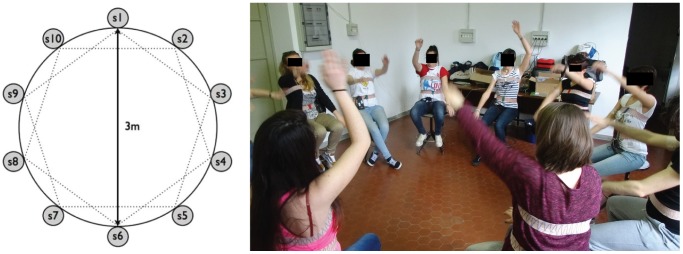
Collective measurement group setup. Left Panel: position of the participants during the study. Right panel: participants of the study during the protocol. The individuals in this manuscript gave written informed consent, as outlined in the PLOS consent form, to participate in the study and to publish their case details. The Ethics committee of the University of Pavia, Italy approved the study protocol.

We tested 5 conditions: 1) *Initial baseline*. We asked the participants to remain quiet and silent for 4 minutes. 2) *Spontaneous movement*. We asked the participants to execute an alternate arm uplifting on the sagittal plane for 2 minutes, without any imposition of rhythm. In order to maintain the naivety of the movement, participants were told that this recording was needed just for “testing” of the proper functioning of the equipment and was not a “real” recording phase. 3) *Music-associated movement*. We asked the participants to perform the same movement as above, while listening to a music track (“Born to be alive” Patrick Hernandez, 1979, 130 bpm) for 2 minutes. Again, we did not give any instruction on whether or how to synchronize the movements with music, similar to spontaneous movement. 4) *Metronome-associated movement*. We asked the participants to perform the same movement as above for 2 minutes, while listening to a metronome rhythm (130 bpm) similar to the music tempo. As for previous conditions, we did not give any instruction on whether or how to synchronize the movements with the metronome. 5) Final baseline. The participants were again asked to remain quiet and silent for 4 minutes. The entire procedure was repeated on the following day with a similar protocol. The order of presentation of conditions 2–4 was randomized, across groups and across repetitions of the protocol on the second day of testing. Each condition was separated by 1-minute break. The tempo of 130 bpm was chosen because, being rather fast, it could suggest different levels of entrainment of movements (1∶2, 1∶4 etc). At the same time it would be just too fast for a 1∶1 synchronization by the subjects with their arms, so it would have been not obvious that this tempo would obtain a perfect synchronization. The particular song was chosen because it employed the desired bpm and because is a well-known piece of music largely used for dance. The participants' placement is shown in Figure 2. On each day, the entire experiment (including instrumentation, familiarization with the laboratory, testing of equipment and recording time) lasted approximately 2 hours.

Testing for the individual measurement group was done on one day, with each participant seated on a chair in a quiet room. No other individuals were present in the room except for the experimenter, who was not visible to the participant throughout the recordings. Individual participants were tested in the same five conditions (except the first individual group in which the final baseline was missing) employed in the collective measurement group, including the same stimuli and the same instructions, with conditions 2–4 presented in random order.

### Data analysis

#### Signal pre-analysis

We first obtained the heart period sequence from the electrocardiogram. This was done by first identifying the peak of the R wave of the ECG in the electrocardiogram, and then constructing the series of the heart period by measuring the R-R interval. This sequence of R-R intervals was converted into a continuous signal at a frequency of 4 Hz by interpolation of the R-R intervals at each data point. All the other signals (thoracic and abdominal respiration, arm's movement) were also directly re-sampled to 4 Hz. Before mathematical analysis, the data underwent linear de-trending of the signals to remove possible baseline drifts of signals.

#### Multivariate analysis of individual participants

A quantitative analysis of the degree of synchronization was done with a multivariate coherence method. In recent years a series of frequency domain approaches were described to assess the relationship (intensity and direction of information flow) between multivariate time series, based on the decomposition of multivariate partial coherences computed from multivariate autoregressive models. New algorithms, called Partial Directed Coherence and Generalized Partial Directed Coherence (GPDC) [16] provide direct structural information for multivariate autoregressive models that simultaneously model many time series [17]. GPDC is considered an improvement of Partial Directed Coherence as it corrected some of its inaccuracies [18] and for this reason it was used in the present study. GPDC is used to find the existence of direct connections between pairs of data sets, but in addition to a simple bi-variate model GPDC includes in the calculation the influence of the n-2 remaining sets. Thus, although GPDC would provide a matrix showing in each panel the coherences between pairs of signals (similar to a simpler bi-variate coherence), it does now in the context of a multivariate model, as GPDC considers all participants simultaneously. Furthermore, GPDC decomposes the interaction of the whole data series into directional components (forward and backward influences). The GPDC and derived methods are based on the concept of Granger causality, which states that if some time series Y(t) contains information in past terms that helps in the prediction of another time series X(t), then Y(t) is said to cause X(t) [19]. Further technical details and rationale for its use can be found in [16]. This method was implemented from the Matlab routines MVAR and MVFREQZ ([20], available for download at: http://biosig.sourceforge.net/index.html). We first constructed for each recording the multivariate data matrix, an array containing the sequences of one signal for each of the 10 subjects. This array was used for the autoregressive Partial Correlation Estimation, with the model order set to 12, the frequency range set to 0–2 Hz and the number of frequencies set to 400 (thus, setting the frequency resolution to 0.005 Hz). The autoregressive Partial Correlation Estimation was obtained by selecting the Nutall-Strand unbiased correlation function [21]. The multivariate autoregressive model parameters were than used to compute GPDC. The generalized partial directed coherence in the frequency domain *f* was defined as: [16]:
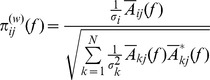
where σ and σ^2^ are the standard deviation and variance of the innovation processes *w*, respectively, *A_kj_ (f)* are the autoregressive terms of pairs k and j of time series (spanning from 1 to N data) and * denotes complex conjugate.

**Figure 3:**
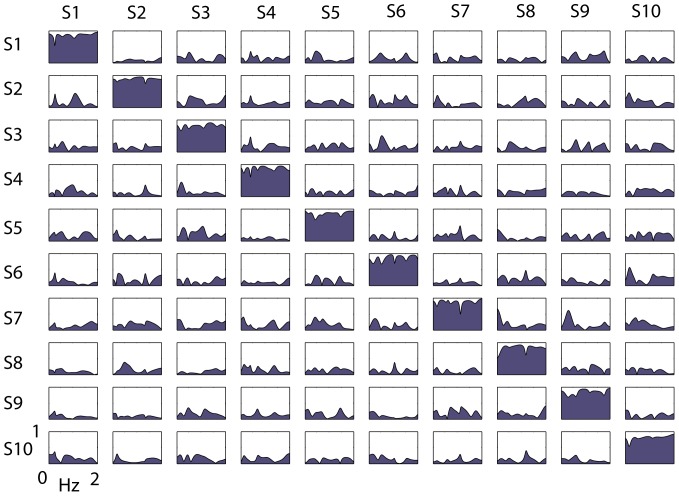
Generalized Partial Directed Coherence (GPDC). Example of multivariate (GPDC) coherence spectra matrix, related to the abdominal respiration signal in the music-associated condition, and in the 10 subjects (S1–S10) of the first collective measurement group on the first day. In each spectrum the abscissa reports the frequency in Hz, and the ordinate reports the coherence, from 0 to 1. Each subject is compared with all the other subjects and with him/herself in the diagonal. Note that the coherence in the diagonal is lower than 1, as each subject is compared with himself, but in the context of a multivariate model that takes into account also the remaining subjects.

Using the data resampled which were obtained in each group of 10 participants and in each of the different conditions of the experiment and for each signal, and applying the GPDC method, we obtained 10×10 matrices of coherence spectra. Figure 3 shows an example, related to the abdominal respiration signal, obtained during listening to music in one collective measurement group on the first day of recording. In the coherence spectra, the reported values span between 0 and 1, where 0 is associated with total asynchrony and 1 with absolute synchronization, thus the greater the value the greater the coordination between the participants in that signal and during that recording. For each of the spectra obtained in each matrix, (except the comparison of each participant with himself), we extracted the average coherence in the low-frequency band (0.035 to 0.15 Hz, LF), in the high-frequency band (0.15 to 0.40 Hz, HF), and in the very-high-frequency band (0.40 to 2 Hz, VHF). These coherence values (obtained from each of the pairs in the 10×10 matrix except for the diagonal) were used for statistical analysis. The LF and HF band are frequently assessed to test the autonomic modulation on heart rate variability [22]. The VHF band was added to test the effects of possible rapid movements. Thus, by dividing the coherence spectra in different bands, we could gain information on the significance of these effects on the autonomic modulation of the heart, and on the effects of breathing and movements on heart rate.

#### Statistic analysis

Values are presented as mean ± standard error of the mean (SEM). Differences due to different conditions, groups and days of recording were evaluated by multivariate analyses of variance (MANOVA). A separate MANOVA was run for each of the four signals (accelerometer, respiration-abdominal, respiration-thoracic, heart period). The coherences on each of three frequency bands (LF, HF and VHF) were treated as dependent variables. The predictors were the Condition (five levels: Baseline1 *vs.* Baseline2 *vs.* Spontaneous movement *vs.* Music-associated movement *vs.* Metronome-associated movement) and the Group membership (two levels: collective *vs.* individual). A second series of MANOVA was run with Condition and Day (two levels: first *vs.* second day) as predictors, to assess the effect of repeating the protocol in the collective group measurement. If overall statistical significance were observed, the Bonferroni post-hoc test was applied to test for differences between conditions. The SPSS software (version 21, Chicago, IL, USA) was used for statistical analysis.

## Results

**Figure 4:**
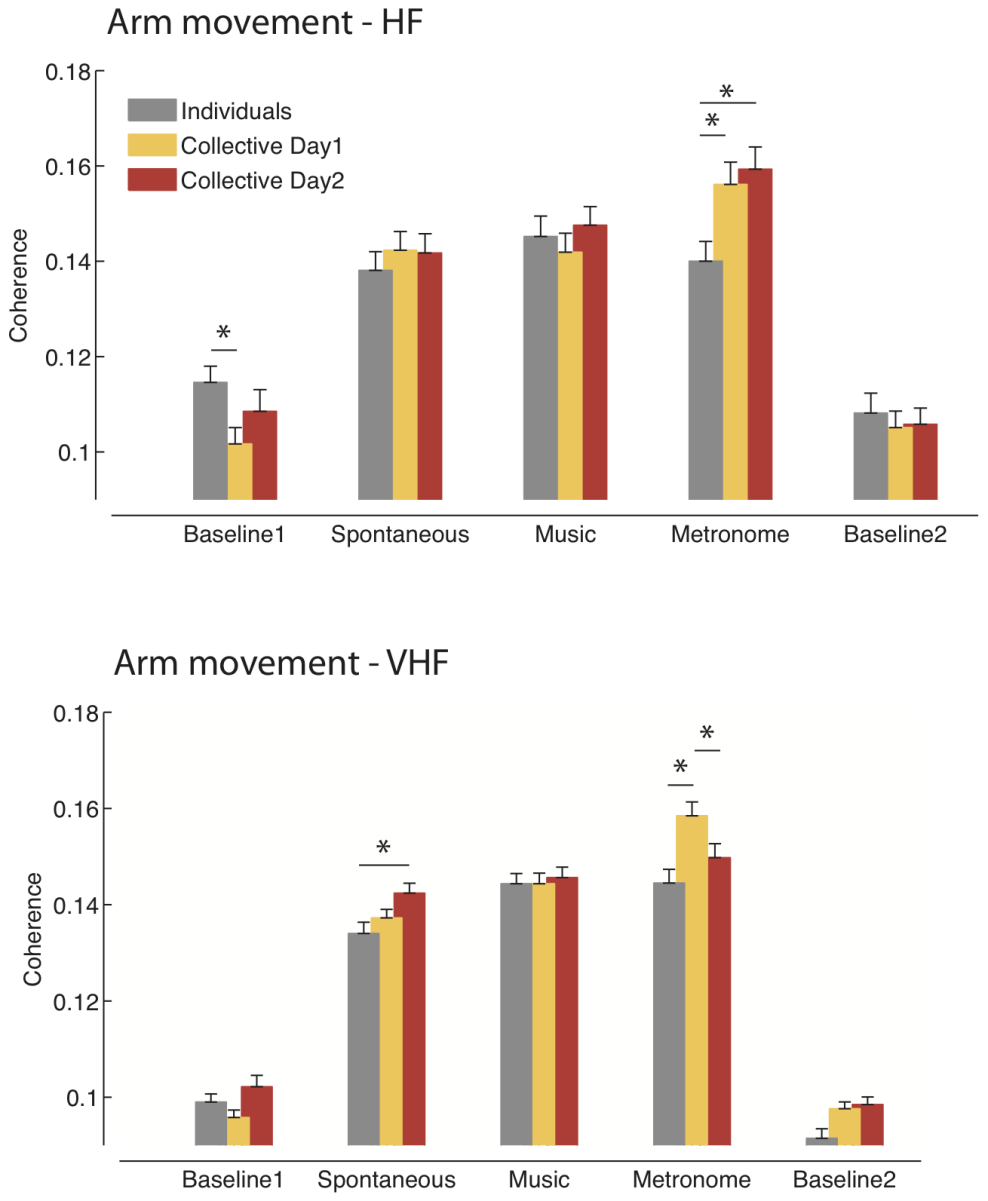
Coherence analysis – Arm movements. The histograms show the degree of synchronization between participants of the arm movement (accelerometer signal), during the five phases of the study: initial resting baseline, spontaneous movement, music-associated movement, metronome-associated movement and final resting baseline. Participants from the individual measurement group are shown in gray; participants from the collective measurement group are shown in colours, with different colours representing the repetition of the protocol over the course of two days. *Top panel*. Results of the coherence in the high frequency band (HF: 0.15–0.4 Hz). Movements were more synchronized in the collective measurement groups for the metronome-associated condition. The greater synchronization of the fluctuations of the accelerometer signal in the resting baseline for the individual measurement groups rules out the possibility that the superior synchronization of the collective in the metronome-associated condition was due to artifacts or differences in the baseline level of the accelerometer signal. *Bottom panel.* Results of the coherence in the very high frequency band (VHF: 0.4–2 Hz). Movements were overall more synchronized in the collective measurement groups than in the individual measurement groups. On the repetition of the protocol, the peak of synchronization previously observed in the metronome-associated condition decreases, while an increase of collective synchronization is observed for the spontaneous, un-cued movements.

**Figure 5:**
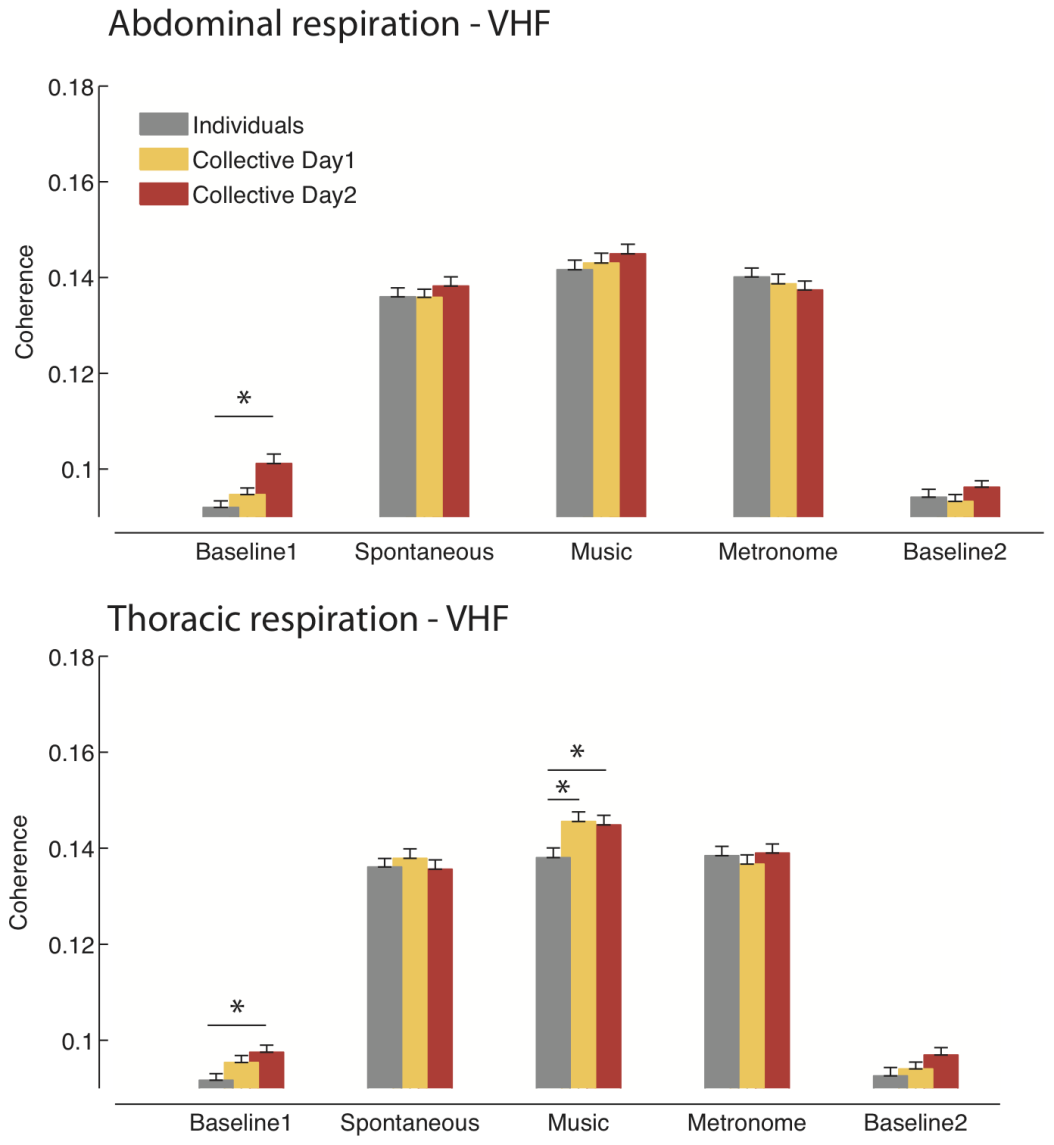
Coherence analysis – Respiration. The histograms show the degree of synchronization between participants for the abdominal (*top panel*) and thoracic (*bottom panel*) respiratory signals, in the very high frequency band (VHF: 0.4–2 Hz), during the five phases of the study (see Figure 4 for details). Participants in the collective measurement group show greater synchronization of respiration than participants in the individual measurement group. This is especially pronounced in the initial resting baseline and in the music-associated condition.

The results of the coherence analysis are shown in Figures 4 and 5. The database with the GPDC scores for each group in each condition is available as Supplementary Information (Database S1).

### Coherence analysis – LF

The analyses of the LF did not reveal significant differences between the collective and individual measurement groups.

### Coherence analysis – HF

#### Arm movements (Figure 4)

Motor coherence emerged in the collective measurement group, significantly higher than in the individual measurement group, for the metronome-associated condition (Condition*Group interaction: F_(4,2582)_  = 3.4, p<0.01; post-hoc: p<0.01). This superiority was not due to differences in the level of the baseline signal: the accelerometer signal recorded during the first resting baseline showed in fact higher coherence for the individual measurement group (p<0.03). Coherence during movements was always higher than the level recorded during the resting baselines (main effect of Condition: F_(4,2582)_  = 44.24, p<0.001), for the collective as well as for the individual measurement groups and with no difference between the spontaneous, music-associated and metronome-associated conditions. The same behaviour was present in the collective measurement group on the second day of testing, which confirmed the superiority of the collective motor coherence over the individuals' in the metronome-associated condition (Condition*Group interaction: F = 2.56_(4,2582)_, p<0.05; post-hoc: p = 0.001).

#### Respiration-abdominal, Respiration-thoracic, Heart period

The analyses of the HF did not reveal significant differences between the collective and individual measurement groups for the respiratory or cardiac signals.

### Coherence analysis – VHF

#### Arm movements (Figure 4)

The VHF revealed a pattern similar to the HF, with motor coherence in the metronome-associated condition being higher in the collective measurement groups than in the individual measurement groups (Condition*Group interaction: F_(4,2582)_  = 4.28, p<0.01; post-hoc: p<0.001). Coherence during movements was always higher than the level recorded during the resting baselines, for the collective as well as for the individual measurement groups (all p<0.001). However, synchronization in the various active conditions differed between the two groups. In the individual measurement groups, the music-associated and metronome-associated conditions yielded a similar degree of synchronization (p>0.9), which in both cases was greater than in the spontaneous movement condition (both p<0.02). Instead, synchronization in the metronome-associated condition for the collective measurement groups exceeded all other conditions (all p<0.001), while spontaneous and music-associated movements resulted in comparable synchronization (p>0.2). The repetition of the experimental protocol on the second day of testing modified the synchronization of the collective measurement groups (Day*Condition interaction: F_(4,2690)_  = 3.39, p<0.01). Metronome-associated synchronization decreased (p<0.01), and a tendency was observed for synchronization during spontaneous movement to increase (p = 0.11). As a result, the collective groups exhibited on the second day an overall greater synchronization than the individuals (main effect of Group: F_(1,2582)_  = 10.55, p = 0.001). Bonferroni corrected t-tests showed that the collective measurement groups on the second day were more synchronized than the individuals especially in the spontaneous movement condition (p<0.02).

#### Respiration-abdominal (Figure 5)

The analyses of the abdominal respiration on the first day of testing did not reveal significant differences between the collective and individual measurement groups. Repeating the protocol on the second day in the collective measurement groups significantly increased respiratory synchronization (main effect of Day: F_(1,2690)_  = 4.32, p<0.04). As a result, the collective groups on the second day of testing exhibited overall greater respiratory synchronization than the individuals (main effect of Group: F_(1,2582)_  = 5.2, p<0.03). The advantage of the collective measurement groups over the individuals was especially marked in the initial baseline (Condition*Group interaction: F_(4,2582)_  = 2.52, p<0.05; post-hoc: p = 0.001), with similar tendencies for the spontaneous and music-associated conditions.

Respiratory synchronization was higher in the active conditions, compared to the baselines, for the collective as well as for the individual measurement groups (all p<0.001). No differences were found in the individual measurement groups between the spontaneous, music-associated and metronome-associated conditions. However, in the collective measurements groups, the music-associated condition yielded a degree of synchronization higher than the metronome-associated condition (p<0.05). No significant differences were found between the initial and the final baseline recordings.

#### Respiration-thoracic (Figure 5)

Thoracic respiration was globally more synchronized in the collective measurement groups than in the individuals (main effect of Group: F_(1,2582)_  = 4.41, p<0.04). Bonferroni corrected t-tests showed that the collective measurement group was more synchronized than the individuals especially in the music-associated condition (p<0.005). The repetition of the experimental protocol on the second day confirmed the overall greater synchronization of the collective groups over the individuals (main effect of Group: F_(1,2582)_  = 7.94, p<0.01). Bonferroni corrected t-tests showed that the collective measurement group on the second day was more synchronized than the individuals not only in the music-associated condition (p = 0.01), but also in the initial baseline condition (p<0.03). No significant differences were found between the initial and the final baseline recordings.

#### Heart period

No differences were found between the collective and individual measurement groups in the cardiac signals.

## Discussion

The present study investigated the synchronization of movement and of autonomic variables within a group of participants performing a simple arm movement. First, we found that performing a cyclical action within a group results in spontaneous motor synchronization between participants. Motor synchronization manifested immediately in the context of a task-irrelevant metronome cue, and extended to an un-cued condition on a repetition of the experimental protocol one day later. Second, we found that collective synchronization is not limited to the movement, but also involves the respiratory rhythms. In fact, participants were found to breathe together while moving the arm in the context of a task-irrelevant music cue. Finally, synchronization of breathing patterns within a group may happen in the absence of shared movement, as participants were found to breathe together even at rest. All these effects were observed despite the fact that participants did not receive explicit instructions about reciprocal coordination or about synchronization with the cue.

### Spontaneous motor synchronization

Influential theories have suggested that behaviour is an emergent consequence of the reciprocal relations that exist between an individual and its environment. This concept can be traced back at least to Gibson's ecological approach to visual perception [23], and has been more recently developed within the theoretical framework of the self-organized dynamical systems [24]. This view has challenged the traditional understanding of behaviour as linear, bottom up and causally determined by an isolated, inner decisional entity. Instead, the organization processes underlying behaviour would be distributed across mind, body and environment, through various physical and informational coupling mechanisms [25]. Consistent with this *embedded* view of action and cognition [26], a wealth of literature has shown that the rhythmical coordination of two oscillators tends to happen in the form of a single synergetic system, or coordinative structure [27]. This has been shown in different contexts such as: within-person rhythmical interlimb coordination [28]; in the coordination that occurs between the rhythmical limb movement of an individual and a visual environmental rhythm [29]; between the rhythmical movements of two interacting individuals [6]. This latter case is especially important because between-individual synchronization has been shown to happen even unconsciously, regardless of whether subjects are intentionally adjusting their behaviours. Previous studies on spontaneous synchronization have mostly studied dyads (e.g., [2]). Only one study to our knowledge has previously investigated whether similar dynamics of spontaneous motor synchronization can occur in a larger group context [30]. These authors recorded the sound of clapping from an audience in a naturalistic setting and found evidence for periodical synchronized clapping. One limitation of this study is that group synchronization was not quantified from the behaviour of each individual, but rather described as a global phenomenon and backed up with sparse observations from isolated participants. A few recent studies have tackled this challenge and have derived synchronization from the movements of each individual within a group setting. However, the presence in these studies of either explicit synchronization instructions ([31], with 6 participants) or shared goals ([32], with 9 participants) did not allow observing spontaneous synchronization dynamics. Compared to these previous investigations, the present study provides two novel contributions. First, it employs for the first time individual-participant recordings in the context of undirected group (10 participants) behaviour. The results provide converging evidence to the findings of [30]: despite participants did not share specific goals and were not given any instruction about synchronization, they spontaneously coupled their movements to a degree significantly higher than participants tested individually. Second, although some recent investigations have explored the synchronization of autonomic variables within a group [13], [33]–[36] this is the first study to simultaneously track motor *as well as* autonomic group synchronization, providing insights about the interactions of these two dimensions in the group dynamic.

### Spontaneous synchronization of respiratory rate

We hypothesized that spontaneous motor synchronization would reverberate beyond the motor domain, to involve the cardiovascular and respiratory rhythms. In agreement with these hypotheses, we found evidence for overall greater synchronization of respiratory rhythms in the collective group than in the participants tested individually. Motor and autonomic synchronization in the collective group showed however a different relationship than expected. In fact, motor and autonomic synchronization occurred relatively independently from one another. For example, motor synchronization peaked in the metronome-associated condition, while respiratory synchronization peaked in the music-associated condition. Moreover, the mere fact of being in a group prompted participants to breathe in a synchronized fashion, even in the absence of movement as in the resting condition. This effect was replicated on the second day of testing, where it emerged even more strongly, possible due to a greater familiarity between the group participants. These results indicate first of all that the respiratory synchronization we have described is not a mere by-product of the arm movement task, and instead depends on some dynamic that is inherent to the group setting. Of interest is also the fact that no differences in the degree of cardiac synchronization were found between the collective groups and the participants tested individually. This suggests that group synchronization was only possible for variables that were to some extent under the participants' control, even if not necessarily in a conscious way. Altogether these results suggest that breathing synchronization was achieved in the collective group through a perception-behaviour link analogue to what has been referred to the *chameleon effect* ([37]; see also [38]–[39]). This term refers to the mimicry of, e.g., postures, mannerisms and facial expressions that happens between individuals, in the absence of participant's awareness and even in the absence of a purposive interaction goal.

These results provide novel insights for understanding the mechanisms of group bonding. Previous studies have shown that motor synchronization is capable of influencing higher-level dimensions of human interaction, such as cooperativeness, pro-social behaviours and reciprocal liking [9], [40]. However, the mechanisms that make possible this influence are still poorly understood. The present investigation supports a possible explanation in terms of synchronized autonomic responses [41]. We have shown that sharing a group situation, either with or without an action associated with it, results in significant synchronization of respiratory rate between the members of a group. This happened despite no common goals or relevant emotional connections still existed between the group members. It is possible to hypothesize that such shared autonomic responses could mediate the previously described feelings of bonding between the people. For example, it has been shown that assuming certain breathing patterns induces correspondent emotional feelings [42], and that imitating another individual's breathing pattern in the absence of vision improves accuracy in identifying the action he/she is engaged in [43]. Furthermore, shared patterns of respiratory function could be a key mechanisms to support the so called “muscular bonding” [44], capable of creating a bridge between the motor and the social aspects of the synchronization dynamics.

Patterns of autonomic synchronization have been previously described for situations of great importance for the individual's feeling of sharing and bonding, such as the mother-infant relationship [45]–[46] or the psychotherapy settings [47]–[48]. All these situations are characterized by the presence of strong emotions, intimacy within the couple and a strive for attunement from (at least) one of the participants. Along a similar venue, a recent study by Konvalinka et al. [15] has shown heart-rate synchronization between the performers and the spectators of a highly emotional fire-walking ritual. In this latter study, the emotional factor and the personal connections were identified as the crucial mechanism mediating synchronization. In fact, autonomic synchronization was observed in the absence of any shared motor activity, but was especially driven by a specific and very short trigger event (the fire-walk). Also, no synchronization with the performer was observed for people that were simply witnessing the show, without any personal involvement. Cardiac and respiratory synchronization was also described in performers during choir singing [13], even if in this latter case the control of breathing was explicit and directly related to the shared task of singing together.

Altogether, these previous and the present investigation converge from different experiments to the autonomic rhythms as an important route for mediating interpersonal synchronization. We therefore propose that synchronization of autonomic functions can be achieved by (at least) two different group dynamics: a) a relatively top-down, triggered by the active emotional engagement in the interpersonal dynamic or b) a relatively bottom-up, that is emotionally neutral and is triggered by involuntary perceptual-behavioural linkages and by shared motor activity. It is interesting to notice that most previous studies that reported autonomic synchronization in the context of emotionally pregnant situations reported synchronization of heart rate, which did not emerge in our study. It is possible to speculate that bottom-up autonomic synchronization would rely on coupling of the breathing in the first place, with heart rate becoming synchronized in a later stage, either due to cardio-respiratory entrainment or due to some emergent emotional quality of the group experience. Alternatively or in addition, the mild exercise performed by the arm movement could have been the cause for reducing the respiratory sinus arrhythmia, i.e. the respiratory-induced variations in heart rate. In fact, it is well known that respiratory sinus arrhythmia markedly drops during exercise [49]. If this were the case, then heart period synchronization could have been missed by the particular experiment performed.

In conclusion, regardless of its top-down or bottom-up origin, autonomic synchronization would provide a physiological base for the experience of group bonding, which possibly would be maximized when both components are present at the same time.

### Cueing effects

Apart from describing inter-subject synchronization, the present investigation was informative about factors that can modulate such synchronization. Motor synchronization was maximal in the presence of the metronome, while synchronization of respiration was enhanced in the music-associated condition. The increase of synchronization in the presence of an external periodic cue is to be expected in the light of a wealth of previous literature showing that external rhythms can entrain movements, favouring the formation of stable behavioural patterns (e.g., [50]–[52]). Also, the rhythmical component of auditory stimuli has been previously shown to entrain autonomic signals [53]–[54]. The differential effect of metronome and music on movement and respiration, respectively, can be explained in the light of the model proposed by Kelso et al. [55]. Entrainment between two rhythmic units happens more easily the smaller the difference in their eigenfrequencies is. Music offers several possible frequencies to entrain to, both relatively fast ones, as the musical beat, as well as relatively slower ones, as the musical phrase. The relatively slow respiratory dynamic is therefore more likely to be entrained in the context of a musical template, which is richer than the metronome in terms of slow frequencies. On the other hand, the simplicity and clarity of the metronome pacing seems best suited to entrain the arm movement dynamic, which is capable of tuning to relatively fast frequencies.

It is especially important to notice that this effect was found despite the fact that participants did not receive any explicit instruction about synchronization with each other or with the metronome. This resembles what happens in several naturalistic situations of group rituals and ceremonies. In these contexts, group synchronization often is not achieved through explicit, top-down instruction on how to precisely behave. Instead, group synchronization emerges as a bottom-up, spontaneous process, with external cues like simple musical rhythms that provide an implicit support.

### Further developments

One direction for further development of this work would involve comparing music and metronomes with different features (e.g., speed, rhythmic complexity, tonal context, etc.) than the one we employed, to understand whether specific background cues could be particularly effective in supporting the emergence of spontaneous group synchronization. It would also be interesting to investigate the subjective dimension of the group experience, for example collecting pre and post scores of appreciation and familiarity within the participants, to use them as covariates in the analysis of synchronization and to investigate changes in group dynamic following the intervention.

### Conclusions

Understanding group dynamics and interaction is becoming a central issue for both social and biological sciences. The present investigation has provided novel data showing motor and respiratory group synchronization that spontaneously emerged as a consequence of being in a group context and performing a simple arm movement. Furthermore, a novel portable equipment capable of obtaining multiple synchronized signals in different people has been described. Complementing previous results that described the importance of emotions and reciprocal bonding in the emergence of synchronization, these results reveal the underlying physiological mechanisms that are potentially responsible for the feeling of group bonding and coordinated human interaction.

## Supporting Information

Database S1 The database contains the coherence data for each pair of participants, in each group and in each experimental condition. Coherence is computed using the GPDC method (Baccala et al., 2007), on the movement, respiratory and cardiac signals. The analyses were focused on the following three frequency bands: “Low frequencies” (LF): 0.035–0.15 Hz; “High frequencies” (HF): 0.15–0.4 Hz; “Very high frequencies” (VHF): 0.4–2 Hz. Condition: 1 =  Initial resting baseline1; 2 =  Final resting baseline; 3 =  Spontaneous movement; 4 =  Metronome-associated movement; 5 =  Music-associated movement. Group: 0 =  Individual measurement group; 1 =  Collective measurement group. Experiment: 1 =  First set of groups; 2 =  Second set of groups; 3 =  Third set of groups. Each set of groups comprises n = 10 participants for the collective measurement and n = 10 participants for the individual measurement. Day: 1 =  First day; 2 =  Second day. Order: 1 =  Condition performed in first position; 2 =  Condition performed in second position; 3 =  Condition performed in third position; 4 =  Condition performed in fourth position; 5 =  Condition performed in fifth position.
(XLS)
